# The Art of War: Ferroptosis and Pancreatic Cancer

**DOI:** 10.3389/fphar.2021.773909

**Published:** 2021-12-10

**Authors:** Jiao Liu, Rui Kang, Daolin Tang

**Affiliations:** ^1^ The Third Affiliated Hospital of Guangzhou Medical University, Guangzhou, China; ^2^ Department of Surgery, UT Southwestern Medical Center, Dallas, TX, United States

**Keywords:** autophagy, ferroptosis, pancreatic cancer, tumorigenesis, targeted therapy

## Abstract

Pancreatic cancer is a devastating gastrointestinal cancer, characterized by late diagnosis, low treatment success rate, and poor survival prognosis. The most common pathological type of pancreatic cancer is pancreatic ductal adenocarcinoma (PDAC), which is mainly driven by the K-Ras oncogene. Ferroptosis was originally described as Ras-dependent cell death, but is now defined as lipid peroxidation-mediated regulated necrosis, accompanied by excessive activation of the autophagy degradation pathway and limited membrane repair capacity. The impaired ferroptotic pathway is involved in many types of cancer, including PDAC. On the one hand, the chronic inflammation caused by ferroptotic damage contributes to the formation of K-Ras-driven PDAC. On the other hand, drug-induced ferroptosis is an emerging strategy to suppress tumor growth in established PDAC. In this mini-review, we outline the core process of ferroptosis, discuss the regulatory mechanism of ferroptosis in PDAC, and highlight some of the challenges of targeting ferroptosis in PDAC therapy.

## Introduction

Pancreatic ductal adenocarcinoma (PDAC) is the most common pathological type of pancreatic cancer, accounting for more than 90% of all pancreatic malignancies (Kleeff et al., 2016). The *KRAS* gene is mutated in approximately 85–90% of PDAC and is the main driver of pancreatic tumorigenesis (Buscail et al., 2020). Despite improvements in surgical techniques, chemotherapy regimens, and the introduction of neoadjuvant chemoradiotherapy or chemoimmunotherapy, PDAC still accounts for 3% of all cancers and 7% of all cancer deaths in the United States (Siegel et al., 2021). Due to modifiable lifestyle factors, such as high-fat diets, the incidence of PDAC is increasing (Heinen et al., 2009). The American Cancer Society estimates that by 2021, there will be 60,430 pancreatic cancer diagnoses and 48,220 deaths in the United States (Siegel et al., 2021). From 2014 to 2021, the general 5-years survival rate of patients with PDAC slowly increased from 6 to 10%. The poor outcomes of PDAC are mainly due to the late diagnosis of the disease and its resistance to treatments involving cell death. Thus, it is essential to understand the cell death machinery of PDAC and to develop new treatment strategies (Chen et al., 2021a). Recent studies have shown that inducing ferroptotic cell death may be an attractive therapy for various types of cancer, including PDAC (Su et al., 2020; Chen et al., 2021b; Shi et al., 2021).

## The Core Mechanism of Ferroptosis

**FIGURE 1:**
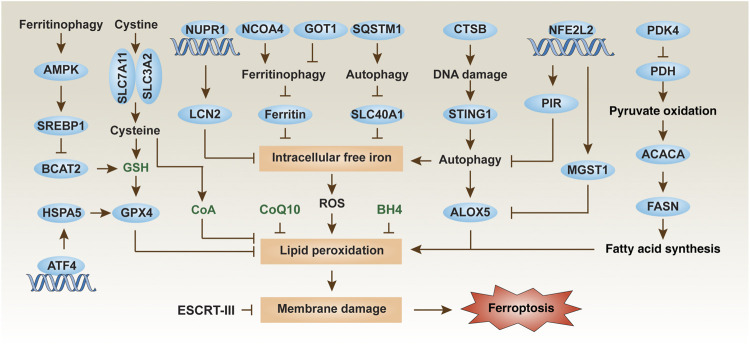
Regulation mechanisms and signaling pathways of ferroptosis in PDAC cells. Ferroptosis is an iron-dependent cell death driven by lipid peroxidation and subsequent membrane damage. The level of ferroptosis in PDAC cells can be regulated in multiple ways, including through autophagic degradation, transcription factors, and metabolic pathways.

The term “ferroptosis” was first proposed to describe a type of iron-dependent non-apoptotic cell death in cancer cells with RAS mutations (Dixon et al., 2012). Today, the core molecular mechanism of ferroptosis is involved in the production of lipid peroxidation and subsequent plasma membrane damage (Stockwell et al., 2017; Tang et al., 2020a). During ferroptosis, reactive oxygen species (ROS) can be obtained from the iron-dependent Fenton reaction, a mitochondrial electron transport chain-mediated reaction, or a membrane NADPH oxidase (NOX)-mediated reaction (Xie et al., 2016). However, the connection between the multiple sources of ROS production during ferroptosis remains obscure. Three antioxidant systems [glutathione (GSH), coenzyme Q10 (CoQ10), and tetrahydrobiopterin (BH4)] have been shown to inhibit ferroptosis caused by oxidative damage (Dixon et al., 2012; Bersuker et al., 2019a; Kraft et al., 2020; Dai et al., 2020a). Among them, the system xc^−^-GSH-glutathione peroxidase 4 (GPX4) axis plays a major role in blocking lipid peroxidation during ferroptosis (Figure 1). System xc^−^, a transmembrane protein complex composed of two subunits, namely solute carrier family 7 member 11 (SLC7A11) and solute carrier family 3 member 2 (SLC3A2), mediates the entry of cystine into cells to exchange glutamate. Once cystine enters cells, it is quickly reduced to cysteine, which is required for GSH synthesis. GSH is a substrate for the antioxidant GPX4 to prevent the accumulation of toxic lipids. GPX4 and SLC7A11 also regulate other types of non-ferroptotic death, indicating that it may not be possible to distinguish them based on a single molecular event (Ran et al., 2006; Canli et al., 2016; Kang et al., 2018; Chen et al., 2021c). Alternatively, apoptosis inducing factor mitochondria associated 2 (AIFM2) plays a GPX4-independent role in limiting ferroptosis by sustaining the production of reduced GSH (Bersuker et al., 2019b; Doll et al., 2019) or increasing membrane repair (Dai et al., 2020a).

Polyunsaturated fatty acids (PUFAs) are the main peroxidation substrates for ferroptosis in cell membranes. Consequently, increasing PUFA synthesis can increase the sensitivity to ferroptosis, which is positively regulated by acyl-coenzyme A (CoA) synthetase long-chain family member 4 (ACSL4) (Dixon et al., 2015; Yuan et al., 2016; Doll et al., 2017; Kagan et al., 2017). Apart from this, the biosynthesis of plasmalogens from peroxisomes also contributes to ferroptosis (Zou et al., 2020a). PUFAs are found in most foods, but are highest in fatty fish, seeds, and nuts. It is possible to adjust the sensitivity to ferroptosis by changing the content and type of dietary fat. Finally, two families of lipid peroxidases [lipoxygenase (ALOX) and cytochrome P450 oxidoreductase (POR)] play a context-dependent role in mediating toxic lipid production during ferroptosis (Yang et al., 2016; Wenzel et al., 2017; Chu et al., 2019; Li et al., 2020; Zou et al., 2020b; Yan et al., 2021). However, the molecular effectors of ferroptosis have not yet been identified. As a conservative membrane repair mechanism, the calcium-dependent endosomal sorting complexes required for transport (ESCRT)-III pathway can be activated to separate damaged membranes in various cancer cells (including PDAC) during ferroptosis (Dai et al., 2020b).

## Ferroptosis in Pancreatic Tumorigenesis

The inflammatory process has become a key mediator of the development and progression of pancreatic cancer. Consistent with this notion, ferroptotic damage can release damage-associated molecular pattern molecules (DAMPs), thereby creating an inflammatory tumor microenvironment for tumor growth and development (Bianchi, 2007). For example, the conditional depletion of *Gpx4* in the pancreas or a high-iron diet accelerates the development of *Kras* ^*G12D*^-driven pancreatic tumors in mice (Dai et al., 2020c). This process is mediated by the release of nuclear DAMP 8-hydroxydeoxyguanosine (8-OH-dG) by ferroptotic cells. The released 8-OH-dG activates the stimulator of interferon response CGAMP interactor 1 (STING1, also known as TMEM173) pathway in surrounding macrophages, thereby inducing the release of cytokines (e.g., interleukin 6) to maintain the chronic inflammatory microenvironment of pancreatic tumorigenesis driven by *Kras* ^*G12D*^ (Dai et al., 2020c). These results explain the basic aspects of the inflammatory tumor microenvironment mediated by ferroptotic death in PDAC. Ferroptotic PDAC cells can also release KRAS^G12D^ protein into the extracellular space, and macrophages take up KRAS^G12D^ protein through advanced glycosylation end-product specific receptor (AGER, best known as RAGE), which eventually leads to macrophage polarization for tumor growth (Dai et al., 2020d). In contrast, the conditional deletion of *Slc7a11* in the pancreas inhibits *Kras* ^*G12D*^ */Tp53* ^*R172H*^ mutation-driven pancreatic tumors in mice (Badgley et al., 2020), suggesting that additional *Tp53* mutations may transform the carcinogenic effects of ferroptotic damage into anticancer effects in *Kras* ^*G12D*^-driven PDAC. In general, these animal studies show that ferroptosis plays a dual role in pancreatic tumorigenesis, depending on gene deletions and mutations. It remains questionable whether genomic instability can produce genetic diversity in driving ferroptosis. It also needs to examine whether *Gpx4* depletion has a similar effect in promoting mutant KRAS-driven tumorigenesis in other cancers, such as colorectal cancer and non-small cell lung cancer.

## Regulation of Ferroptosis in PDAC

The regulator of PDAC is involved in multiple molecules (Table 1). We discussed them from the following three perspectives, although this classification is rough considering the observed diversity of molecular mechanisms of ferroptosis.

**TABLE 1:** Main regulators of ferroptosis in PDAC.

Name	Function	Mechanism	Refs.
NCOA4	Promoter of ferroptosis	Induce autophagic degradation of ferritin	Hou et al. (2016)
SQSTM1	Promoter of ferroptosis	Induce autophagic degradation of SLC40A1	Li et al. (2021a)
STING1	Promoter of ferroptosis	Induce autophagy-dependent ferroptosis	Li et al. (2020)
ALOX5	Promoter of ferroptosis	Induce lipid ROS production	Li et al. (2020)
CTSB	Promoter of ferroptosis	Induce DNA damage and lysosomal dysfunction	Kuang et al. (2020), Nagakannan et al. (2021)
ACACA	Promoter of ferroptosis	Increase fatty acid synthesis	Song et al. (2021)
FASN	Promoter of ferroptosis	Increase fatty acid synthesis	Song et al. (2021)
PDH	Promoter of ferroptosis	Increase pyruvate oxidation	Song et al. (2021)
AMPK	Promoter of ferroptosis	Inhibit BACT2 expression	Wang et al. (2020)
SREBP1	Promoter of ferroptosis	Inhibit BACT2 expression	Wang et al. (2020)
SLC7A11	Repressor of ferroptosis	Increase GSH or CoA synthesis	Badgley et al. (2020), Zhang et al. (2019), Badgley et al. (2020)
GOT1	Repressor of ferroptosis	Inhibit autophagic degradation of ferritin	Kremer et al. (2021)
SLC40A1	Repressor of ferroptosis	Promote iron export	Li et al. (2021a)
Ferritin	Repressor of ferroptosis	Promote iron storage	Hou et al. (2016)
ATF4	Repressor of ferroptosis	Induce HSPA5 expression	Zhu et al. (2017)
HSPA5	Repressor of ferroptosis	Inhibit GPX4 degradation	Zhu et al. (2017)
GPX4	Repressor of ferroptosis	Inhibit lipid ROS production	Zhu et al. (2017), Liu et al. (2021a)
PDK4	Repressor of ferroptosis	Inhibit pyruvate oxidation	Song et al. (2021)
BCAT2	Repressor of ferroptosis	Increase GSH synthesis	Wang et al. (2020)
MTOR	Repressor of ferroptosis	Inhibit autophagy-dependent ferroptosis	Liu et al. (2021a)
NFE2L2	Repressor of ferroptosis	Inhibit expression of antioxidant gene	Kuang et al. (2021), Hu et al. (2021)
MGST1	Repressor of ferroptosis	Inhibit oxidative stress	Kuang et al. (2021)
PIR	Repressor of ferroptosis	Inhibit oxidative DNA damage	Kuang et al. (2021)
POLG	Repressor of ferroptosis	Inhibit mitochondrial DNA damage-dependent autophagy	Li et al. (2020)
TFAM	Repressor of ferroptosis	Inhibit mitochondrial DNA damage-dependent autophagy	Li et al. (2020)
NUPR1	Repressor of ferroptosis	Increase LCN2 expression	Liu et al. (2021b)
LCN2	Repressor of ferroptosis	Promote iron export	Liu et al. (2021b)
ESCRT-III	Repressor of ferroptosis	Inhibit membrane repair	Dai et al. (2020b)

## Degradation Systems

Macroautophagy (hereafter autophagy) and the ubiquitin-proteasome system are the two degradation systems responsible for regulating cellular homeostasis (Li et al., 2021b). Depending on the substrate being degraded, autophagic pathways play a significant role in pancreatic ferroptosis. A significant recent advance is that the autophagic degradation of the iron storage protein ferritin (a process also called ferritinophagy) (Hou et al., 2016) or the iron transporter solute carrier family 40 member 1 (SLC40A1, also known as ferroportin-1) (Li et al., 2021a) can increase the accumulation of free iron in cells, thereby inducing the Fenton reaction to produce ROS for ferroptosis in PDAC cells (Figure 1). More recently, glutamic-oxaloacetic transaminase 1 (GOT1) inhibition promotes ferroptosis in PDAC by inducing ferritinophagy to initiate iron-dependent oxidative damage (Kremer et al., 2021). Nuclear receptor coactivator 4 (NCOA4) (Hou et al., 2016) and sequestosome 1 (SQSTM1) (Li et al., 2021a) function as autophagy receptors to recognize and degrade ferritin or SLC40A1, respectively, during ferroptosis. However, identifying specific autophagy cargo receptors for ferroptosis remains a challenge.

In addition to the classic ferroptosis activators (erastin and RSL3), zalcitabine (a drug used to treat human immunodeficiency virus infection) can cause mitochondrial damage, thereby activating STING1-dependent autophagy pathway and inducing ALOX5-related ferroptotic death in human PDAC cells (Li et al., 2020). The activation and release of cystatin B (CSTB, a lysosomal cysteine protease) can partially act as a mediator of ferroptosis by amplifying the STING1 pathway in human PDAC cells, arguing that ferroptosis is a form of autophagy-dependent lysosomal cell death coupled with a DNA sensor pathway (Kuang et al., 2020; Nagakannan et al., 2021).

In addition to autophagy, other degradation pathways also regulate ferroptosis by affecting the stability of GPX4 protein. For example, transcription factor 4 (ATF4)-mediated heat shock protein family A (Hsp70) member 5 (HSPA5) expression related to endoplasmic reticulum stress can prevent the degradation of GPX4, thereby increasing the ferroptosis resistance of PDAC cells (Zhu et al., 2017). In contrast, high-dose rapamycin can induce ferroptosis by promoting the degradation of GPX4 (Liu et al., 2021a). Because autophagy is generally used as a pro-survival pathway in PDAC, the induction of autophagy-dependent ferroptosis may provide a way to kill established PDAC cells (Görgülü et al., 2020). Nevertheless, clinically available autophagy inhibitors (e.g., chloroquine) may weaken the anticancer activity of ferroptosis activators (Li et al., 2021a).

## Metabolic Pathways

In the 1920s, Otto Warburg discovered that even in the presence of oxygen (aerobic glycolysis), cultured tumor cells had a high rate of glucose uptake and glycolysis (Vander Heiden et al., 2009). This Warburg effect triggers metabolic abnormalities, thereby promoting tumor growth or causing treatment resistance to transitional drugs (e.g., gemcitabine). Indeed, hyperglycemia occurs frequently in most patients with pancreatic cancer and is associated with a poor prognosis. Unexpectedly, ferroptosis in PDAC cells induced by system xc^−^ inhibitors (erastin and sulfasalazine), but not GPX4 inhibitors (RSL3 and FIN56), requires high-glucose conditions (Song et al., 2021). In contrast, high-glucose limits staurosporine-induced cell death (Song et al., 2021). These results imply that glucose selectively confers susceptibility to ferroptosis, rather than apoptosis. In line with this notion, diabetes induced by a high-fat diet in mice also increased the anti-PDAC activity of ferroptosis inducers (Song et al., 2021).

Subsequent studies of metabolic mechanisms showed that pyruvate oxidation, but not pyruvate reduction, in mitochondria promotes ferroptosis in PDAC cells by activating acetyl-CoA carboxylase alpha (ACACA) and fatty acid synthase (FASN)-mediated fatty acid synthesis and subsequent ALOX5-dependent lipid peroxidation (Figure 1) (Song et al., 2021). This pro-ferroptosis process caused by glucose is negatively regulated by pyruvate dehydrogenase kinase 4 (PDK4), a repressor of pyruvate oxidation in mitochondria by blocking pyruvate dehydrogenase (PDH) (Song et al., 2021). An integrated metabolic reprogramming pathway may drive the production of fatty acid for ferroptosis.

In addition to glucose and lipids, amino acids also affect ferroptosis. For example, branched chain amino acid aminotransferase 2 (BCAT2) can inhibit ferroptosis in PDAC cells that is induced by system xc^−^ inhibitors (erastin, sorafenib, and sulfasalazine) by producing sulfur amino acid for GSH synthesis (Wang et al., 2020). Moreover, system xc^−^-mediated cystine input is beneficial to the biosynthesis of CoA, which plays a GSH-independent role in preventing IKE-induced ferroptosis in PDAC cells (Badgley et al., 2020). These findings provide a feedback mechanism for controlling ferroptosis through amino acid metabolism. Although the role of AMP-activated protein kinase (AMPK)-sterol regulatory element binding transcription factor 1 (SREBP1) pathway in ferroptosis is related to the type of cancer, the activation of this signaling pathway by ferritinophagy limits the expression of BCAT2 in PDAC cells (Wang et al., 2020). Since AMPK is an important kinase in various metabolic pathways, targeting the AMPK pathway combined with ferroptosis induction may be a strategy worthy of further exploration (Song et al., 2018; Lee et al., 2020). Recently, the endogenous metabolite itaconate induces iron death in PDAC cells by activating ferritinophagy (Qu et al., 2021), highlighting the new metabolic pathway of ferroptosis.

## Stress Sensors

Another important research direction is to identify and study redox sensors in ferroptosis. Nuclear factor, erythroid 2-like 2 (NFE2L2, best known as NRF2) is a transcription factor that is sensitive to the redox state of cells under various cell death stimuli. NFE2L2 is negatively regulated by Kelch-like ECH-associated protein 1 (KEAP1), which targets NFE2L2 for protein degradation by the ubiquitin-proteasome system. In response to the stimulation of ferroptosis, autophagy receptor SQSTM1 binds and inhibits KEAP1, thereby promoting the activation of NFE2L2 and increasing the expression of antioxidant genes (Anandhan et al., 2020). Specifically, the NFE2L2-targeted genes microsomal glutathione S-transferase 1 (MGST1) (Kuang et al., 2021) and pirin (PIR) (Hu et al., 2021) have recently been identified as redox-sensitive repressors of ferroptosis in PDAC cells by binding ALOX5 or limiting the oxidative damage of DNA-mediated autophagy, respectively (Figure 1). These studies provide new insight into the complex mechanisms of NFE2L2-dependent redox signaling in PDAC. A key unanswered question is whether certain types of cell death are particularly associated with dysregulated NFE2L2 signaling.

In addition to NFE2L2, other stress-related transcription factors are also involved in the defense of ferroptosis. Nuclear protein 1, transcriptional regulator (NUPR1) is upregulated in the pancreas during various stresses, including ferroptotic damage (Liu et al., 2021b). As a pro-survival response, NUPR1-mediated expression of the iron exporter lipocalin 2 (LCN2) can prevent iron accumulation, thereby limiting oxidative damage and ferroptosis in PDAC cells (Figure 1) (Liu et al., 2021b). In a xenograft mouse model, the NUPR1 inhibitor ZZW-115 enhances the tumor suppressor effect of the ferroptosis activator IKE (Liu et al., 2021b). Together, targeting antioxidant transcription factors can enhance ferroptosis-mediated therapy in PDAC, although downstream effectors may be diverse.

## Opportunities and Challenges

The past few years have witnessed a rapid explosion of studies on ferroptosis and various cancers, including PDAC. Because PDAC responds weakly to all current treatment regimens, targeting the ferroptotic pathway may provide an alternative approach for this lethal disease. Exciting preclinical studies have shown that several drugs [artesunate (Eling et al., 2015) and zalcitabine (Li et al., 2020)] can suppress PDAC by inducing ferroptosis, although they may have off-target effects. Several therapy regimens related to ferroptosis (e.g., gemcitabine + sulfasalazine, sorafenib + sulfasalazine) have also been explored in PDAC in animal models. The ultimate goal of research is to develop clinically available drugs for the modulation of the ferroptosis pathway that can kill PDAC alone or in combination with other drugs. The challenge in developing effective drugs that induce ferroptosis is not to cause unnecessary side effects and to target specific sites in ferroptotic pathway. Under treatment selection pressure, resistance to treatment may appear due to the expansion of pre-existing subclonal populations or the evolution of drug-resistant cells (Yang et al., 2014; Hangauer et al., 2017; Viswanathan et al., 2017). Although there are currently no clinical trials of ferroptosis-dependent treatment strategies, it is still necessary to establish the relationship between molecular characteristics and response to specific drugs in preclinical studies.

In addition to understanding the processes and functions of ferroptosis in the context of the ever-evolving complex cell death network, we need to develop powerful ferroptotic biomarkers in humans (Chen et al., 2021d). Since inflammation is a double-edged sword, overcoming the immune side effects of ferroptotic damage may require a deeper understanding of the interaction between tumor cell death and immune cells. Several studies in other cancers have shown that DAMP released during erastin-induced ferroptosis may activate adaptive tumor immunity to inhibit tumor growth (Efimova et al., 2020; Tang et al., 2020b). However, the occurrence of ferroptosis in dendritic cells or CD8^+^ T cells can impair their anti-tumor function (Han et al., 2021; Ma et al., 2021). In the next few years, the impact of genetic mutations, degradation pathways, and metabolic plasticity on susceptibility to ferroptosis in the different component cells of the tumor microenvironment will be an active area of research (Chen et al., 2021e; Chen et al., 2021f; Tang et al., 2021).
